# A Combined Two-mRNA Signature Associated With PD-L1 and Tumor Mutational Burden for Prognosis of Lung Adenocarcinoma

**DOI:** 10.3389/fcell.2021.634697

**Published:** 2021-01-26

**Authors:** Congkuan Song, Zhiquan Wu, Qingwen Wang, Yujin Wang, Zixin Guo, Sheng Li, Weidong Hu

**Affiliations:** ^1^Department of Thoracic Surgery, Zhongnan Hospital of Wuhan University, Wuhan, China; ^2^Hubei Key Laboratory of Tumor Biological Behaviors and Hubei Cancer Clinical Study Center, Wuhan, China; ^3^Department of Thoracic Surgery, People’s Hospital of Qichun County, Huanggang, China; ^4^Department of Biological Repositories, Zhongnan Hospital of Wuhan University, Wuhan, China; ^5^Human Genetics Resource Preservation Center of Hubei Province, Wuhan, China

**Keywords:** lung adenocarcinoma, immunotherapy, prognosis, tumor mutational burden, PD-L1

## Abstract

Due to biological heterogeneity, lung adenocarcinoma (LUAD) patients with the same stage may exhibit variable responses to immunotherapy and a wide range of outcomes. It is urgent to seek a biomarker that can predict the prognosis and response to immunotherapy in these patients. In this study, we identified two genes (ANLN and ARNTL2) from multiple gene expression data sets, and developed a two-mRNA-based signature that can effectively distinguish high- and low-risk patients and predict patients’ response to immunotherapy. Furthermore, taking full advantage of the complementary value of clinical and molecular features, we combined the immune prognostic signature with clinical features to construct and validate a nomogram that can predict the probability of high tumor mutational burden (>10 mutations per megabyte). This may improve the estimation of immunotherapy response in LUAD patients, and provide a new perspective for clinical screening of immunotherapy beneficiaries.

## Introduction

As a malignant tumor with high morbidity and mortality, lung cancer poses a serious threat to human life and health. Lung adenocarcinoma (LUAD) is the most common histological subtype of lung cancer (Chen et al., 2019), and has its unique biological characteristics. In clinical practice, the TNM staging system is most commonly used to predict the prognosis of LUAD. The later the stage, the worse the prognosis. However, the existence of tumor biological heterogeneity may also make this prognostic system, which only depends on inherent anatomical information (tumor size, lymph node, and distant metastatic status), unable to accurately predict disease progression and prognosis of these patients. Therefore, it is necessary to continue to search for reliable biomarkers that can predict patient outcomes. In recent years, the rapid rise of immunotherapy has completely changed the treatment mode of LUAD. Patients who do not benefit from conventional surgery, chemotherapy, or radiation are clearly benefiting from this emerging treatment (Brahmer et al., 2015; Hellmann et al., 2018). However, the majority of patients are still lack of response to immunotherapy in clinic (Rooney et al., 2015; Sunshine and Taube, 2015; Li et al., 2016), which is a long way from meeting clinical needs. Thus, immunotherapy-related biomarkers are particularly crucial for screening patients with non-small cell lung cancer (NSCLC) who benefit. At present, programmed cell death protein-1 (PD-1)/programmed cell death-ligand 1 (PD-L1) pathway inhibitors have achieved significant efficacy in immunotherapy for lung cancer, and PD-L1 has become the most commonly used predictive marker for immune checkpoint inhibitors (ICIs) of lung cancer. In 2015, Food and Drug Administration (FDA) approved Pembrolizumab as a first-line treatment for patients with PD-L1 expression ≥50% and no clear driving gene EGFR and ALK mutations. The objective response rate (ORR) was 44.98% and the disease stability time was 10.3 months in these patients receiving immunotherapy, both of which were significantly better than chemotherapy (D’Incecco et al., 2015). Since then, many studies (Zheng et al., 2014; Herbst et al., 2016; Reck et al., 2016) have confirmed the reliability of PD-L1 as a predictor of immunotherapy. Besides, a large number of studies (Teng et al., 2015; Yarchoan et al., 2017; Hause et al., 2018) have proved that tumor mutational burden (TMB), microsatellite instability (MSI), tumor lymphocytes (TIL), and others can be used as biomarkers for predicting the response to immunotherapy. Several studies (Goodman et al., 2017; Yarchoan et al., 2017; Hellmann et al., 2018; Singal et al., 2019) have shown that the higher TMB is, the easier it is supposed to be recognized by immune cells, and the higher the response to immunotherapy is. TMB is a type of biomarker which has been explored and developed continuously. It may serve to choose the patients who are suitable for immunotherapy. Given this, our study attempts to find the genes closely related to PD-L1 and TMB from multiple gene expression data sets, to explore biomarkers that can accurately predict the prognosis and immunotherapy response of LUAD. Furthermore, we also explored the immune-infiltrating cells and molecular mechanisms of prognostic differences in LUAD. Taking full advantage of the complementary value of clinical and molecular features, we combined the immune prognostic signature with clinical features to develop a nomogram that can predict the probability of high TMB to improve the estimation of immunotherapy response in LUAD patients.

## Materials and Methods

### Data Download and Processing

From the data portal of The Cancer Genome Atlas (TCGA)^1^ as of 11 June 2020, we downloaded transcriptome data, tumor mutational data, and corresponding clinical data of 535 LUAD tumor tissues and 59 lung normal tissues. As an independent external validation dataset, mRNA expression data and corresponding clinical information were also downloaded from GSE31210 (Okayama et al., 2012) in the GEO database^2^. This data set contains clinical information for 226 cases with LUAD and transcriptome data for 226 lung tumor tissues and 20 normal lung tissues. These cases without detailed survival information were excluded from this study.

### Identification of Differentially Expressed Genes

Using the “limma” package in R (Ritchie et al., 2015), we performed the differential expression analysis of LUAD tumor tissues and normal tissues in both datasets (TCGA and GSE31210). The threshold values were |log_2_ FC (fold-change)| > 1 and adj.*P*-value < 0.05.

### Identification of Genes Associated With PD-L1 Expression and Prognosis

Further, we analyzed the correlation between differentially expressed genes (DEGs) and PD-L1 expression in both datasets (| Pearson correlation coefficients| > 0.3 and *p*-value < 0.001). To identify prognosis-related genes, we performed univariate Cox analysis of these genes positively associated with PD-L1 expression. These genes with *p*-value less than 0.05 were considered to affect the prognosis of LUAD patients.

### Construction and Verification of the Signature

We took the intersection of prognosis-related genes in both datasets. Next, the intersection genes were analyzed by multivariate Cox analysis (stepwise model) in TCGA train set (random sampling of 50% data from TCGA entire set). Akaike information criterion (AIC) was used to avoid over-fitting. Genes with the highest likelihood ratio and lowest AIC values were eventually selected. At the same time, coefficients of these genes were assessed. Based on gene expression values and coefficients, we calculated the risk score for each sample by the following formula (Sullivan et al., 2004):

$$\text{riskScore}=\sum_{i=1}^{n}\mathrm{Coefficienti}\times \mathrm{Expressioni},$$

**FIGURE 1:**
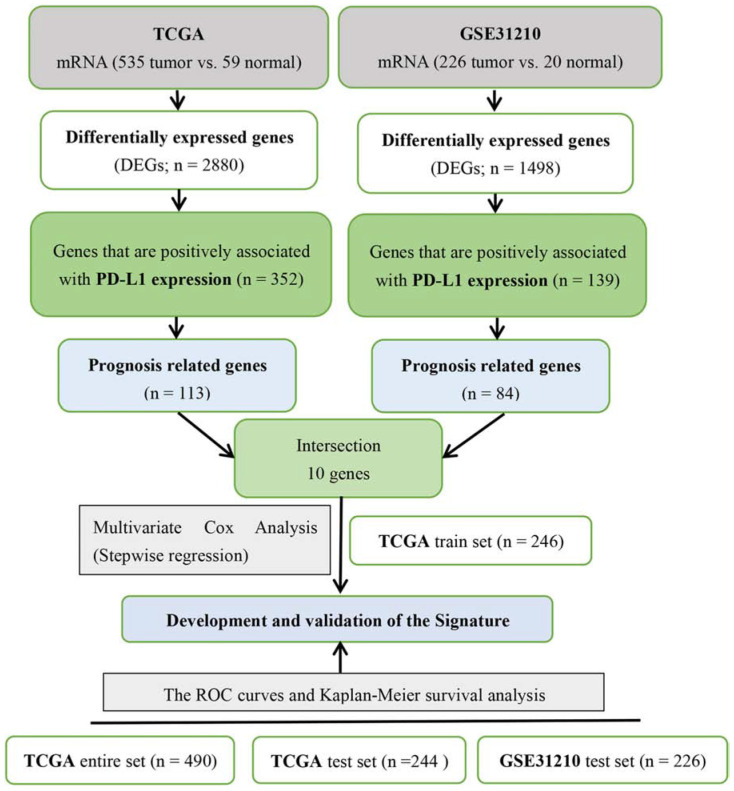
Detailed process of the combined two-mRNA signature construction.

With the median risk score as the cut-off point, we divided patients into high- and low-risk groups. Next, we used the data from four datasets (TCGA entire set, TCGA train set, TCGA test set and GSE31210 test set) to verify the accuracy of the signature by Kaplan–Meier survival curves and ROC curves. The detailed process of the signature construction is shown in Figure 1.

### Mutation Distribution and Related Transcription Factors of the Intersection Genes

By accessing the cBioPortal program^3^, we inputed the intersection genes to obtain their mutation distribution in LUAD tumor tissues. In addition, we obtained the transcription factor (TF) subset from the Cistrome^4^ and corresponding TF expression values from the TCGA database. Through correlation analysis, we identified TFs associated with the intersection genes (|Pearson correlation coefficients| >0.3 and *p*-value < 0.001).

### Expression of Signature Genes at mRNA and Protein Levels and Their Relationship to Survival

Based on the TCGA entire set, we compared the expression of signature genes in LUAD tumor and normal tissues and their relationship to survival. To further explore the expression of signature genes at protein level, we visited Human Protein Atlas (HPA)^5^. In addition, the relationship between signature genes and clinical features was further revealed.

### Evaluation of Immune-Infiltrating Cells in LUAD

To assess LUAD immune-infiltrating cells, we used the Cell type identification by estimating relative subsets of RNA transcripts (CIBERSORT; Newman et al., 2015) to determine the aggregation of 22 immune-infiltrating cells in LUAD samples in the TCGA database and to explore the correlation between signature genes and these immune-infiltrating cells in high- and low-risk patients.

### Gene Set Variation Analysis

Gene set variation analysis (GSVA) is an unsupervised approach to gene set enrichment for a simple population to assess pathway activity variation, primarily on 50 marker pathways described in the Molecular Signatures Database (MSigDB), where each pathway-associated gene set is pruned to contain only unique genes to reduce pathway overlap and pathway redundancy, with most genomes retaining 70% of the associated genes. To explore the differences in metabolic pathways between high- and low-risk patients, GSVA was performed using the “GSEABase” package in R.

### Development and Validation of a Nomogram for Predicting the Probability of High TMB

We obtained tumor mutation data of LUAD from TCGA database. 10 mutations per megabyte (MB) was used as the threshold of high and low TMB (Hellmann et al., 2018; Lv et al., 2019). A high TMB level was defined as >10 mutations per MB, and below that threshold was a low TMB level. Firstly, multivariate logistic regression was performed on clinical characteristics and risk score for 490 patients with LUAD. Variables with *p* values less than 0.05 were re-incorporated into the logistic regression model. Using the “rms” package in R, we constructed a nomogram that could predict the probability of high TMB. Using a bootstrap method with 1,000 resamples, we validated the prediction performance of the nomogram by calculating C-index, AUC value, and drawing ROC and calibration curves. Furthermore, clinical decision curve analysis (DCA) was also used to evaluate the clinical utility of the nomogram.

### Statistical Analysis

All categorical variables were expressed as number (percentage). The Wilcoxon test was performed to compare differences between groups of continuous data. The relationships between signature genes and risk score and immune-infiltrating cells were determined by the Spearman’s correlation analysis. The Kaplan–Meier method was applied to plot survival curves. Survival curves were compared with the log-rank test. All statistical analysis was conducted in R software 3.6.0. A *p*-value less than 0.05 was considered statistically significant.

## Results

### Acquisition of Candidate Genes

**FIGURE 2:**
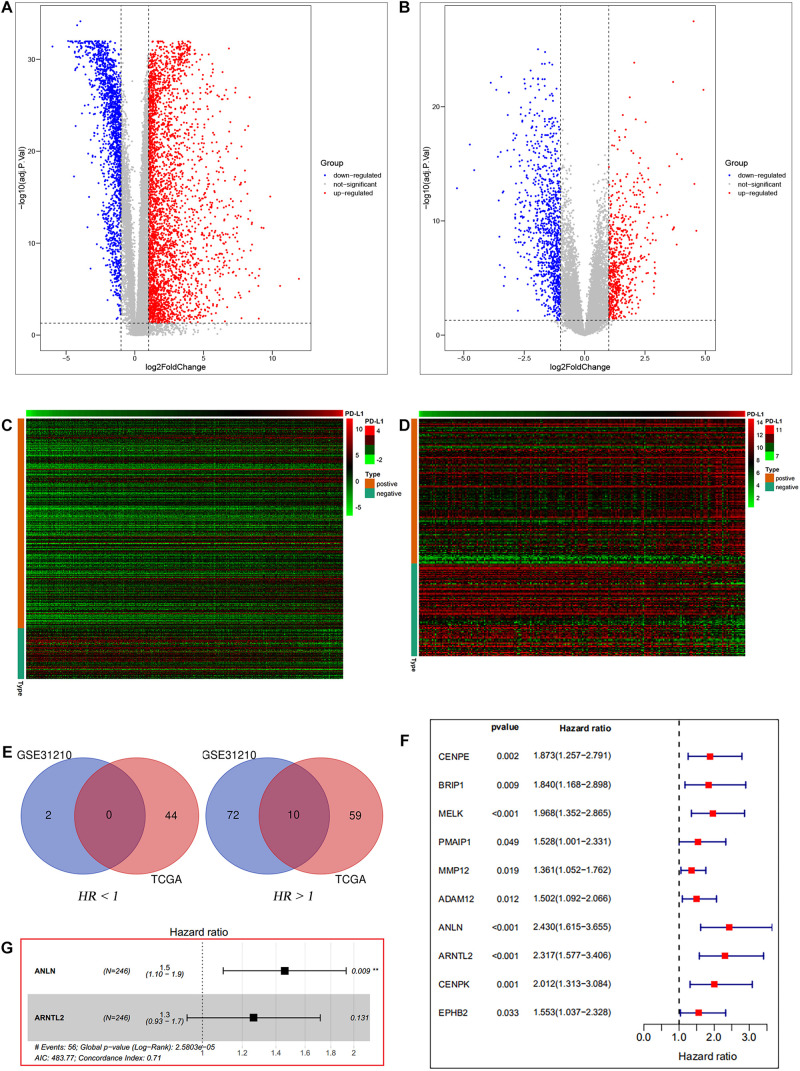
Data filters. Differential expression analysis between lung tumor and normal tissues based on TCGA database **(A)** and GSE31210 dataset **(B)**. PD-L1 correlation analysis based on TCGA database **(C)** and GSE31210 dataset **(D)**. The intersection of prognosis-related genes in the two datasets **(E)**. Univariate Cox analysis of the intersection genes of the two datasets based on TCGA entire set **(F)**. Multivariate Cox analysis of the intersection genes of the two datasets based on TCGA train set **(G)**.

Differential expression analysis was performed on mRNA expression data of LUAD tumor tissues and normal tissues. We obtained 2,880 and 1,498 DEGs from TCGA database and GSE31210 dataset, respectively. Among the 2,880 genes in the TCGA database, there were 1,980 up-regulated genes and 900 down-regulated genes. Of the 1,498 genes in the GSE31210 dataset, 602 were up-regulated and 896 were down-regulated. Volcano maps of differential expression analysis in two databases are, respectively, shown in Figures 2A,B. Afterward, we assessed the correlation between DEGs and PD-L1 expression in the two datasets. Among the 437 genes associated with PD-L1 expression in the TCGA data set, 352 were positively correlated and 85 were negatively correlated (Figure 2C). Among 228 genes associated with PD-L1 expression in GSE31210 data set, 139 genes were positively correlated and 89 were negatively correlated (Figure 2D). Next, we performed univariate Cox analysis on genes positively correlated with PD-L1 expression in the two datasets. There were 113 prognosis-related genes in TCGA dataset, of which 69 were high-risk genes (HR > 1) and 44 were low-risk genes (HR < 1). There were 84 prognosis-related genes in GSE31210 dataset, of which 82 were high-risk genes (HR > 1) and 2 were low-risk genes (HR < 1). Finally, a total of 10 genes (MELK, ADAM12, CENPE, EPHB2, PMAIP1, BRIP1, ANLN, MMP12, CENPK, and ARNTL2) were identified as high-risk genes in both datasets that could affect the prognosis of patients (Figures 2E,F).

### Establishment and Evaluation of the Prognosis Signature

**FIGURE 3:**
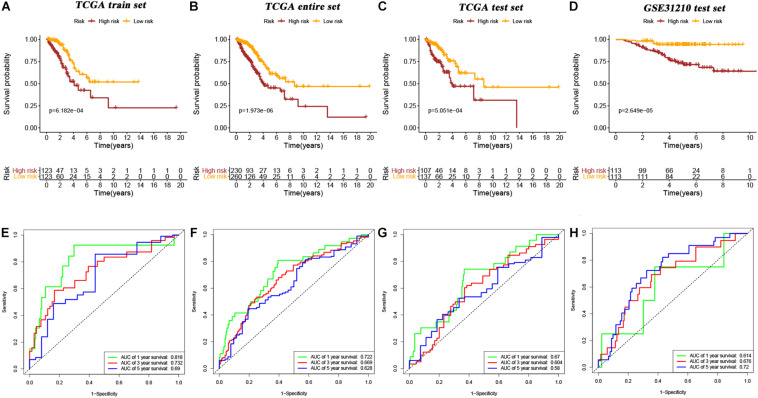
Kaplan–Meier survival curves of OS in high-risk patients vs low-risk patients based on TCGA train set **(A)**, TCGA entire set **(B)**, TCGA test set **(C)**, and GSE31210 test set **(D)**. The ROC curves of the signature for predicting OS in the TCGA train set **(E)**, TCGA entire set **(F)**, TCGA test set **(G)**, and GSE31210 test set **(H)**.

Ten candidate prognosis-related genes were included in multivariate Cox analysis. Eventually two genes (ANLN and ARNTL2) were identified in the signature according to their β coefficients (Figure 2G). Based on the expression values and β coefficients of the two genes, risk score was evaluated by the following formula: Riskscore=(0.377×Expression***_*ANLN*_***)+(0.235×Expression***_*ARNTL2*_***). Patients were divided into high- and low-risk groups with the median risk score as the cut-off point. As shown in Figures 3A–D, high-risk patients showed a worse prognosis than low-risk patients in the four datasets (TCGA entire set, TCGA train set, TCGA test set, and GSE31210 test set; log-rank test, all *p* value < 0.001). Clinicopathological features of patients in the four datasets are shown in Table 1. In the TCGA train set (*n* = 246), the 1-year, 3-year, and 5-year AUC values of the risk model were 0.818, 0.732, and 0.690, respectively, (Figure 3E). In the TCGA entire set (*n* = 490), the 1-year, 3-year, and 5-year AUC values of the risk model were 0.722, 0.669, and 0.628, respectively, (Figure 3F). Moreover, in both the TCGA test set (*n* = 244) and the GSE31210 test set (*n* = 226), the corresponding ROC had high AUC values (Figures 3G,H). This indicated that the signature constructed in this study had excellent predictive performance, and could accurately distinguish high- and low-risk patients.

**TABLE 1:** Basic clinicopathological features of patients in the four datasets.

Variables	TCGA entire set (*n* = 490)	TCGA train set (*n* = 246)	TCGA test set (*n* = 244)	GSE31210 test set (*n* = 226)
**Age**				
<=65	231 (47.1)	119 (48.4)	112 (45.9)	176 (77.8)
>65	249 (50.8)	121 (49.2)	128 (52.5)	50 (22.2)
Missing	10 (2.1)	6 (2.4)	4 (1.6)	–
**Sex**				
Female	262 (53.4)	134 (54.4)	128 (52.5)	121 (53.5)
Male	228 (46.6)	112 (45.6)	116 (47.5)	105 (46.5)
**T stage**				
T1&T2	426 (86.9)	207 (84.2)	219 (89.8)	–
T2&T3	61 (12.4)	37 (15.0)	24 (9.8)	–
Tx	3 (0.7)	2 (0.8)	1 (0.4)	–
**N stage**				
N0	317 (64.7)	153 (62.2)	164 (67.3)	–
N1&N2&N3	162 (33.1)	89 (36.2)	73 (29.9)	–
Nx	11 (2.2)	4 (1.6)	7 (2.8)	–
**M stage**				
M0	324 (66.2)	163 (66.3)	161 (66.0)	–
M1	24 (4.8)	9 (3.6)	15 (6.1)	–
Mx	142 (29.0)	74 (30.1)	68 (27.9)	–
**TNM stage**				
I&II	378 (77.2)	186 (75.6)	192 (78.7)	226 (100.0)
III&IV	104 (21.2)	55 (22.4)	49 (20.1)	–
Unknown	8 (1.6)	5 (2.0)	3 (1.2)	–
**EGFR mutation**				
Yes	79 (16.1)	44 (17.9)	35 (14.3)	127 (56.1)
No	186 (37.9)	85 (34.6)	101 (41.4)	99 (43.9)
Unknown	225 (46.0)	117 (47.5)	108 (44.3)	–
**Status**				
Alive	372 (75.9)	190 (77.2)	182 (74.5)	191 (84.5)
Dead	118 (24.1)	56 (22.7)	62 (25.5)	35 (15.5)
**Risk score**				
High risk	230 (46.9)	123 (50.0)	107 (43.8)	113 (50.0)
Low risk	260 (53.1)	123 (50.0)	137 (56.2)	113 (50.0)

### Applicability of the Constructed Signature in Different Clinical Subgroups

**FIGURE 4:**
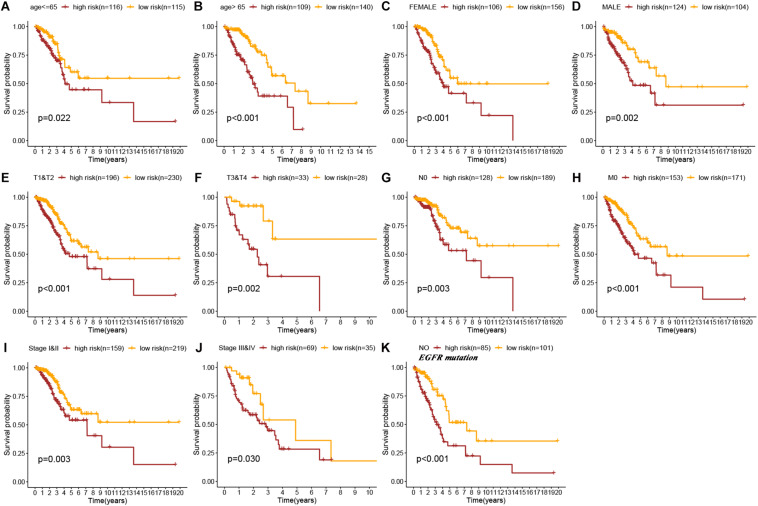
Kaplan–Meier survival curves of OS in high-risk patients vs low-risk patients based on the different LUAD subgroups [age ≤ 65 years **(A)**, age > 65 years **(B)**, female **(C)**, male **(D)**, stage T1&T2 **(E)**, stage T3&T4 **(F)**, stage N0 **(G)**, stage M0 **(H)**, stage I&II **(I)**, stage III&IV **(J)**, and No EGFR mutation **(K)**].

To explore the applicability of the constructed signature in different LUAD populations, subgroup analysis was performed. As shown in the Kaplan–Meier curves, except stage N1-N3, stage M1, and EGFR mutation subgroups, all other subgroups showed that high-risk patients had a worse prognosis than low-risk patients (age < = 65 years old, *p* = 0.022; age > 65 years old, *p* < 0.001; female, *p* < 0.001; male, *p* = 0.002; stage T1–T2, *p* < 0.001, stage T3–T4, *p* = 0.002; stage N0, *p* = 0.003; stage M0, *p* < 0.001; stage I–II, *p* = 0.003; stage III–IV, *p* = 0.030; EGFR-wild, *p* < 0.001, log-rank test; Figures 4A–K). Therefore, the immune prognostic signature was applicable in most LUAD subgroups.

### Mutations Distribution of the 10 Intersection Genes and Their Related TFs

**FIGURE 5:**
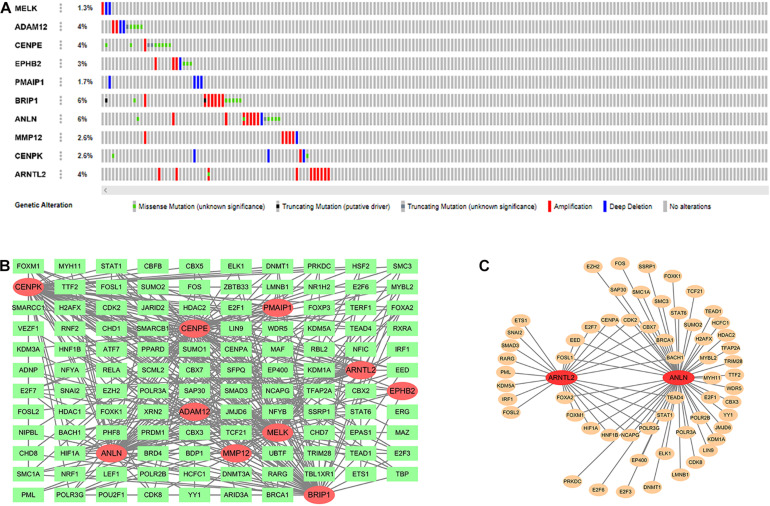
Mutation distribution of 10 intersection genes **(A)**. Ten intersection genes and their related transcription factors (TFs; **B**). Two signature genes and their related TFs **(C)**.

In the cBioPortal database (see text footnote 3), we obtained the mutation distribution of 10 intersection genes in LUAD tissues through the input of gene symbol, as shown in Figure 5A. This figure showed the specific mutation status and proportion of each gene. A subset of TFs was obtained from the Cistrome database (see text footnote 4). Correlation analysis found that of these 10 genes, the most TFs (*n* = 79) were involved in CENPE expression, followed by BRIP1 (*n* = 64). Their interaction network is presented in Figure 5B. In addition, Figure 5C showed the interaction between two signature genes (ANLN and ARNTL2) and corresponding TFs. Among them, ANLN expression was affected by 52 TFs (45 positive and 7 negative), and ARNTL2 expression was influenced by 24 TFs (21 positive and 3 negative; Supplementary Table S1).

### Expression and Survival of Signature Genes at mRNA and Protein Levels

**FIGURE 6:**
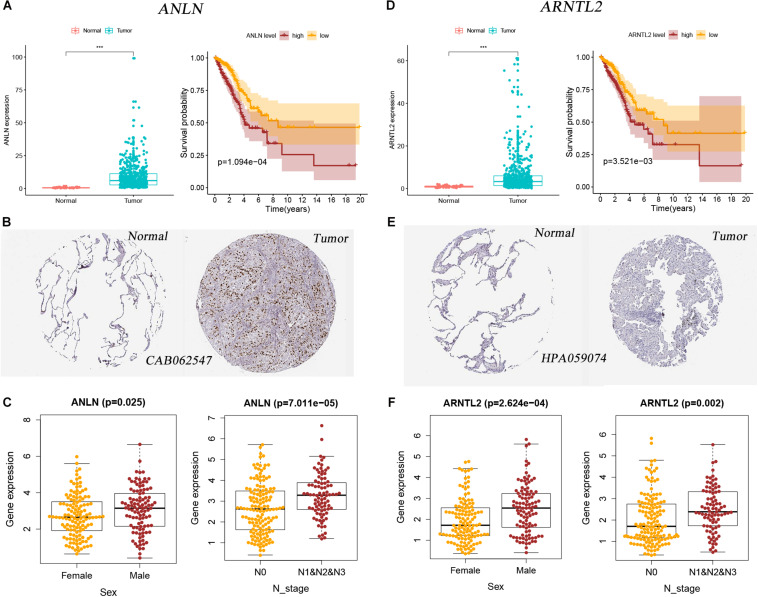
Two signature genes’ expression and their relationship with clinical factors and patients’ outcomes. **(A)** Expression difference of ANLN between tumor and normal tissues and its relationship with survival in the mRNA level. **(B)** Expression difference of ANLN between tumor and normal tissues in the protein level. **(C)** The relationship between ANLN expression and sex and N stage. **(D)** Expression difference of ARNTL2 between tumor and normal tissues and its relationship with survival in the mRNA level. **(E)** Expression difference of ARNTL2 between tumor and normal tissues in the protein level. **(F)** The relationship between ARNTL2 expression and sex and N stage.

Expression differences of ANLN and ARNTL2 between LUAD tumor tissues and normal tissues are shown in Figures 6A,D. Both genes were highly expressed in tumor tissues. At the protein level, the difference is still visible (Figures 6B,E). And the Kaplan–Meier survival curves of both genes showed that poor outcomes were associated with high expression status of genes (log-rank test, all *p* < 0.001; Figures 6A,D). In addition, we explored the relationship between the expression of these two genes and clinical traits. Interestingly, the expression of both genes was significantly correlated with gender and lymph node status. The expression levels of ANLN and ARNTL2 were higher in men and patients with lymph node metastasis (Figures 6C,F).

### The Relationship of Signature Genes and Risk Score With Immune Checkpoints and TMB

**FIGURE 7:**
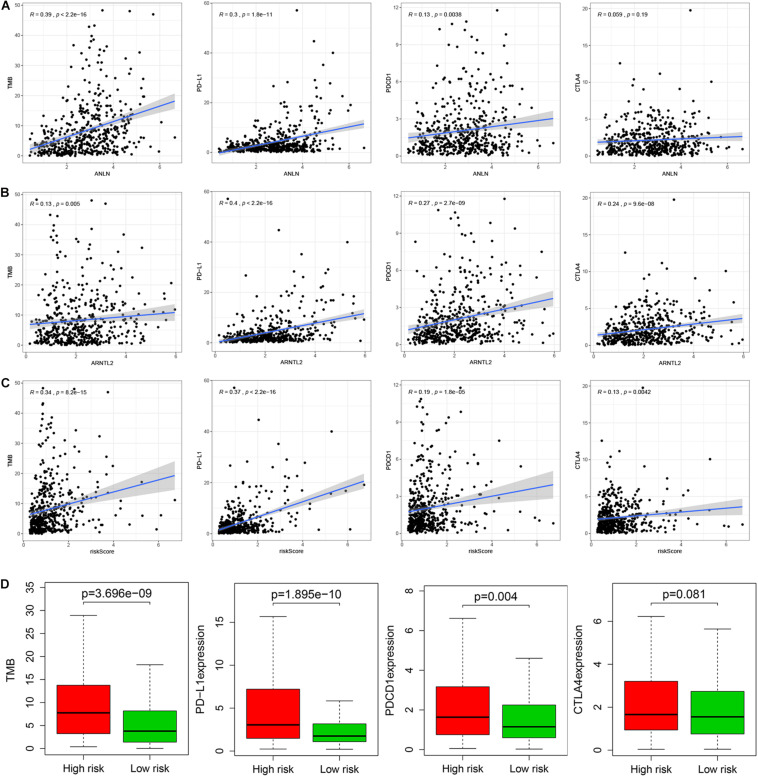
Relationship between ANLN and immune checkpoints (PD-L1, PD-1, and CTLA-4) and TMB **(A)**. Relationship between ARNTL2 and immune checkpoints (PD-L1, PD-1, and CTLA-4) and TMB **(B)**. Relationship between risk score and immune checkpoints (PD-L1, PD-1, and CTLA-4) and TMB **(C)**. Differences in immune checkpoints (PD-L1, PD-1, and CTLA-4) and TMB between high- and low-risk patients **(D)**.

Immune checkpoints (PD-1, PD-L1, and CTAL4) and TMB can better predict patients’ response to immunotherapy. This study found that ANLN and risk score were strongly positively correlated with the expression of TMB and PD-L1, while weakly correlated with the expression of PD-1 and CTLA4 (Figures 7A,C). ARNTL2 was strongly positively correlated with PD-L1 and weakly correlated with TMB (*R* = 0.13, *p* = 0.005), PD-1 (*R* = 0.27, *p* < 0.001), and CTLA4 (*R* = 0.24, *p* < 0.001; Figure 7B). Furthermore, we also compared the differences in immune checkpoints and TMB between high- and low-risk patients, and found that the expression level of CTLA4 in high-risk patients was slightly higher than that in low-risk patients, with no statistical difference (Wilcoxon test, *p* = 0.081). While TMB, PD-1, and PD-L1 levels of high-risk patients were significantly higher than those of low-risk patients (Wilcoxon test, all *p* < 0.001; Figure 7D).

### Tumor Immune Landscape and Pathway Enrichment

**FIGURE 8:**
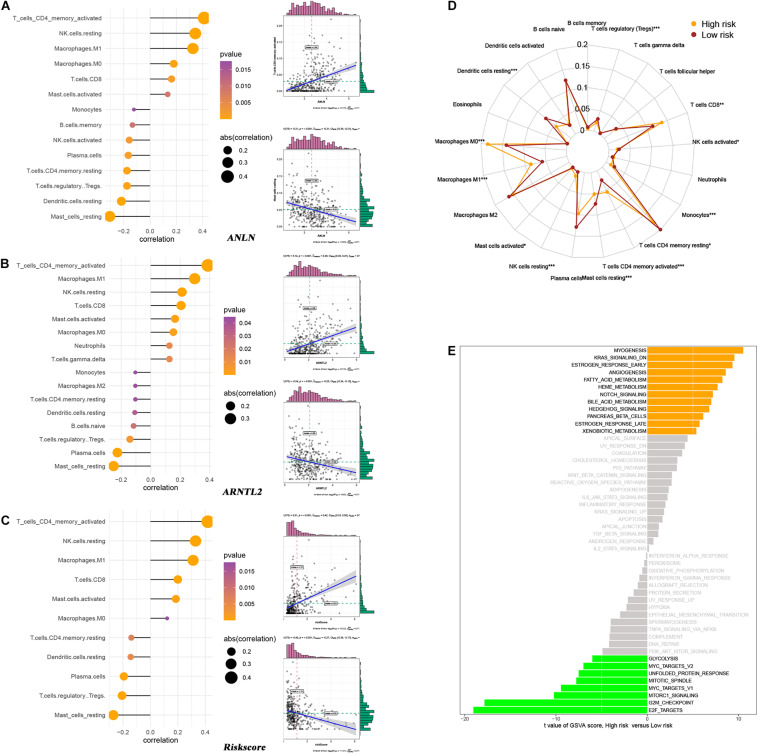
Relationship between ANLN and immune-infiltrating cells **(A)**. Relationship between ARNTL2 and immune-infiltrating cells **(B)**. Relationship between risk score and immune-infiltrating cells **(C)**. Differences in immune-infiltrating cells between high- and low-risk patients **(D)**. Differences in metabolic pathways between high- and low-risk patients **(E)**.

As a widely proposed computational algorithm, CIBERSORT can be used to predict the content of 22 immune-infiltrating cells in each LUAD tumor tissue. Based on this, Spearman’s correlation analysis was used to explore the relationship between ANLN, ARNTL2, risk score and immune-infiltrating cells (Figures 8A–C). It was found that ANLN, ARNTL2 and risk score were strongly positively correlated with T cells CD4 memory activated, and negatively correlated with Mast cell resting. Also, tumor immune landscape of high- and low-risk patients were significantly different (Figure 8D and Supplementary Figure S1). Dendritic cells resting, NK cells resting, Mast cells resting, etc. in tumor tissues of high risk patients were significantly infiltrated, while low risk patients were dominated by T cells CD8, Macrophages M0, Macrophages M1. In addition, GSVA was performed to reveal the differences in metabolic pathways between high- and low-risk patients. The results showed that pathways such as “Myogenesis,” “K-ras Signaling Down,” “Estrogen Response Early,” etc. were more active in high-risk patients, while pathways such as “E2F Targets,” “G2M Checkpoint,” and “mTORC1 signaling” were more enriched in low-risk patients (Figure 8E).

### Predictive Performance of the Established Nomogram

**FIGURE 9:**
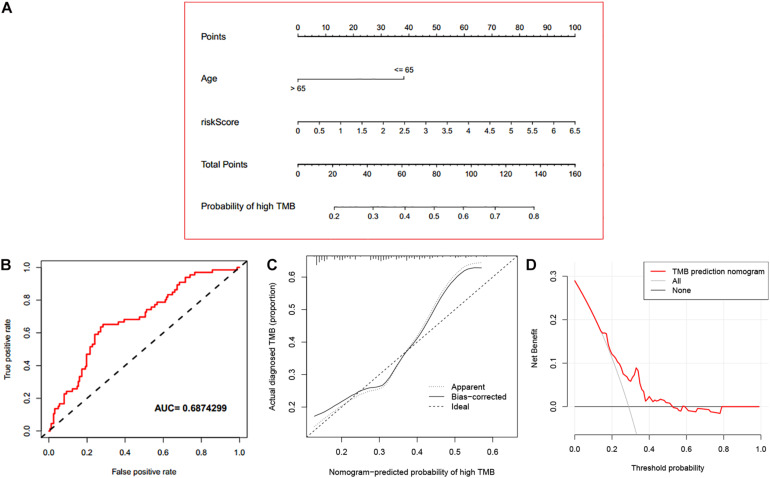
Evaluation of the established nomogram. **(A)** An immune nomogram for predicting the probability of high TMB (>10 mutations per MB) in LUAD patients. **(B)** The ROC curve of the established nomogram. **(C)** The calibration curve of the established nomogram. **(D)** DCA curve of the established nomogram.

Based on the assessed risk score and some clinical features, multivariate logistic regression was performed to construct a nomogram that accurately predicts the probability of high TMB (>10 mutations per MB) for LUAD. Age, and risk score were considered independent predictors of LUAD high TMB (Table 2), which were incorporated into the nomogram. From the nomogram (Figure 9A), we could see that risk score contributed the most to the nomogram total score. The ROC of the constructed nomogram (AUC = 0.689) is illustrated in Figure 9B, and the corresponding calibration curve (Figure 9C) indicated that the TMB predicted by the model was in good agreement with the actually observed TMB. In addition, when the threshold probability was between 0.14 and 0.52, the net benefit of the applied model was better (Figure 9D). This suggested that the established nomogram had good clinical practicability.

**TABLE 2:** Multivariate logistic regression analysis of the probability of high TMB.

Variables	Multivariate analysis			Selected factors for model		
		
	Coef	OR (95% CI)	*P*-value	Coef	OR (95% CI)	*P*-value
Age (>=65 vs <65)	–0.949	0.387 (0.204–0.715)	0.003	−0.919	0.399 (0.215–0.724)	<0.001
Sex (male vs female)	0.293	1.341 (0.718–2.501)	0.355			
T stage (T3&T4 vs T1&T2)	0.526	1.693 (0.701–4.028)	0.235			
N stage (N1&N2&N3 vs N0)	–0.338	0.713 (0.324–1.519)	0.388			
TNM_stage (III&IV vs I&II)	–0.422	0.655 (0.249–1.668)	0.381			
EGFR_mutation (no vs yes)	0.086	1.090 (0.444–2.793)	0.853			
EGFR_mutation (unknown vs yes)	0.227	1.254 (0.539–3.079)	0.607			
RiskScore	0.397	1.487 (1.094–2.044)	0.012	0.370	1.448 (1.084–1.960)	0.013

*Coef, coefficient; OR, Odds ratio; and CI, confidence interval.*

## Discussion

At present, the prognostic prediction of LUAD patients mainly depends on the TNM staging system. In fact, even if the patients are at the same stage, their prognosis may be different, and the response to one same treatment varies. A genomic landscape study (Skoulidis and Heymach, 2019) has reported that this phenomenon may be due to genomic heterogeneity. Mechanically classifying such patients into the same stage will inevitably affect the prognosis of patients and clinical decision-making. Therefore, in the era of precise medicine, a reliable prognostic model of LUAD is urgently needed. PD-L1 and TMB have become biomarkers for predicting response to ICIs in patients with LUAD. In this study, 10 mRNAs related to PD-L1 expression and prognosis were selected from two gene expression data sets. An immune prognostic signature combining two mRNAs was finally constructed. This novel signature could effectively identify patients with high- and low-risk, and be well applied in different clinical subgroups. Moreover, TMB, PD-1, and PD-L1 levels of high-risk patients were significantly higher than those of low-risk patients, which indicated that high-risk patients were more likely to benefit from immunotherapy. Thus, in clinical practice, we only need to measure the expression values of two mRNAs (ANN and ARNTL2) in the tumor tissue of the patient to obtain the patient’s risk score, which in turn can predict the patient’s prognosis and response to immunotherapy. This is bound to help clinicians better judge the prognosis of patients, and then specify more reasonable treatment strategies.

To further explore the potential mechanism of the difference in prognosis between high- and low-risk patients, we analyzed gene set variation and immune infiltrating cells. GO gene sets variation analysis revealed that pathways such as “Myogenesis,” “K-ras Signaling Down,” “Estrogen Response Early” were more active in high-risk patients, while pathways such as “E2F Targets,” “G2M Checkpoint,” and “mTORC1 signaling” were more enriched in low-risk patients. Although the two signature genes in our study were previously shown to identify as potential biomarkers (Luo et al., 2020; Wang et al., 2020), differences in metabolic pathways between high- and low-risk patients based on the established signature have not been previously reported in LUAD, indicating that the two signature genes and varied gene sets or pathways in GSVA have the potential to be further investigated. Additionally, some studies (Budhu et al., 2006; Galon et al., 2006, 2007) have proved that all kinds of cells, cytokines and chemokines that interact with tumor cells in the tumor microenvironment, especially immune cells, were increasingly considered to play vital roles in the body’s anti-tumor activities. In this study, there were significant differences in tumor immune landscape between high- and low-risk patients. Two signature genes (ANLN and ARNTL2) and risk score were strongly positively correlated with T cells CD4 memory activated, and negatively correlated with Mast cell resting. The killing effect of CD4^+^ T cells on tumor is mainly mediated by IFN-g-dependent mechanism. Hung et al. (1998) have reported that activated CD4^+^ T cells may induce delayed type hypersensitivity and attract inflammatory cells, such as macrophages, neutrophils, eosinophils, and NK cells to tumor cells. Tumor infiltrating macrophages will be lost with the deletion of CD4T cells, and the tumor cannot be protected. This suggests that CD4^+^ T cells could directly activate macrophages in lymph nodes or tumor tissues. Besides, there were also significant differences in the infiltration level of other immune-infiltrating cells between high- and low-risk patients, indicating that tumor microenvironment was complex, highly heterogeneous and evolving with tumor development.

Several studies (Rizvi et al., 2015; Carbone et al., 2017) have proved that gene mutation was closely related to the effect of immunotherapy, which has been widely accepted by clinical workers and researchers. A retrospective study from Goodman et al. (2017) analyzed 1,638 tumor patients with TMB quantitative results before immunotherapy, and found that high TMB was independently related to immune response. Similarly, a randomized controlled trial of Pembrolizumab by Rizvi et al. (2015) showed that non-synonymous mutation was related to the improvement of ORR and progression free survival (PFS). Moreover, these studies of Checkmate-032 and Checkmate-227 also confirmed the predictive value of TMB detection in immunotherapy (Antonia et al., 2016; Hellmann et al., 2017). As a popular role in the era of precision medicine, ICIs still need one or a set of clear efficacy prediction indicators to promote their clinical application, while the expression level of tumor PD-L1 still cannot be the absolute standard of immunotherapy guidance. TMB, as another effective predictive biomarker for immunotherapy and independent to PD-L1 expression, has a positive linear correlation between its level and immunotherapy efficacy. In recent years, several targeted sequencing panels have been developed to effectively determine TMB, which requires patients to be in a position to provide a certain amount of tissue samples. However, the limited amount of tumor DNA obtained by conventional or fine-needle biopsy may make the evaluation of TMB challenging, or even impossible, because large sequencing panels are required large amounts of tumor DNA. Given this, combining the mRNA-based signature and clinical factors related to TMB, we developed a nomogram that could individually predict the probability of high TMB in patients. AUC and calibration curve showed that the nomogram had good prediction performance, and its clinical practicability was also confirmed in DCA. Therefore, in clinical work, we might be able to predict the prognosis of patients and determine the probability of high TMB in patients by measuring the expression values of the two signature genes to infer the patient’s risk score. This will undoubtedly contribute to the clinical screening of immunotherapy beneficiaries. This nomogram is expected to be used routinely in the future.

Indeed, this study has some limitations. Although this study used massive cohorts from the TCGA and GEO databases to develop and validate this signature, it has yet to avoid the bias brought by the nature of retrospective research. In addition, the two signature genes identified in this study still need further functional researches to clarify their role in the occurrence and development of LUAD.

To the best of our knowledge, this study is the first to identify and verify the immune prognostic signature containing two genes (ANLN and ARNTL2) in patients with LUAD, which can be used as biomarkers for predicting the prognosis and immunotherapy efficacy of LUAD patients. Moreover, this nomogram combining the two-mRNA signature and age can predict the probability of high TMB (>10 mutations per MB) in LUAD patients, and provide a new clinical application for LUAD in screening the beneficiaries of immunotherapy.

## Data Availability Statement

The datasets presented in this study can be found in online repositories. The names of the repository/repositories and accession number(s) can be found in the article/Supplementary Material.

## Ethics Statement

These data from the SEER database are open to the public for research purposes. This study was also approved by the Institutional Research Committee of Zhongnan Hospital of Wuhan University.

## Author Contributions

CS, ZW, SL, and WH designed the study. CS reviewed relevant literature and drafted the manuscript. ZW participated in the conception and design of this study, as well as the collation and analysis of data. CS, ZW, and QW conducted all statistical analyses. All authors read and approved the final manuscript.

## Conflict of Interest

The authors declare that the research was conducted in the absence of any commercial or financial relationships that could be construed as a potential conflict of interest.

## Footnotes

1 https://portal.gdc.cancer.gov/

2 https://www.ncbi.nlm.nih.gov/geo/

3 https://www.cbioportal.org/

4 http://cistrome.org/

5 https://www.proteinatlas.org/

## Supplementary Material

Supplementary Figure 1 Differences in immune-infiltrating cells between high- and low-risk patients.

Supplementary Table 1 Relationship between signature genes and transcription factors.
