# Supplementary figures and images for: A Combined Two-mRNA Signature Associated With PD-L1 and Tumor Mutational Burden for Prognosis of Lung Adenocarcinoma
Source: Front Cell Dev Biol. 2021 Jan 26;9:634697. doi: 10.3389/fcell.2021.634697 (PMC7875126; doi:10.3389/fcell.2021.634697)

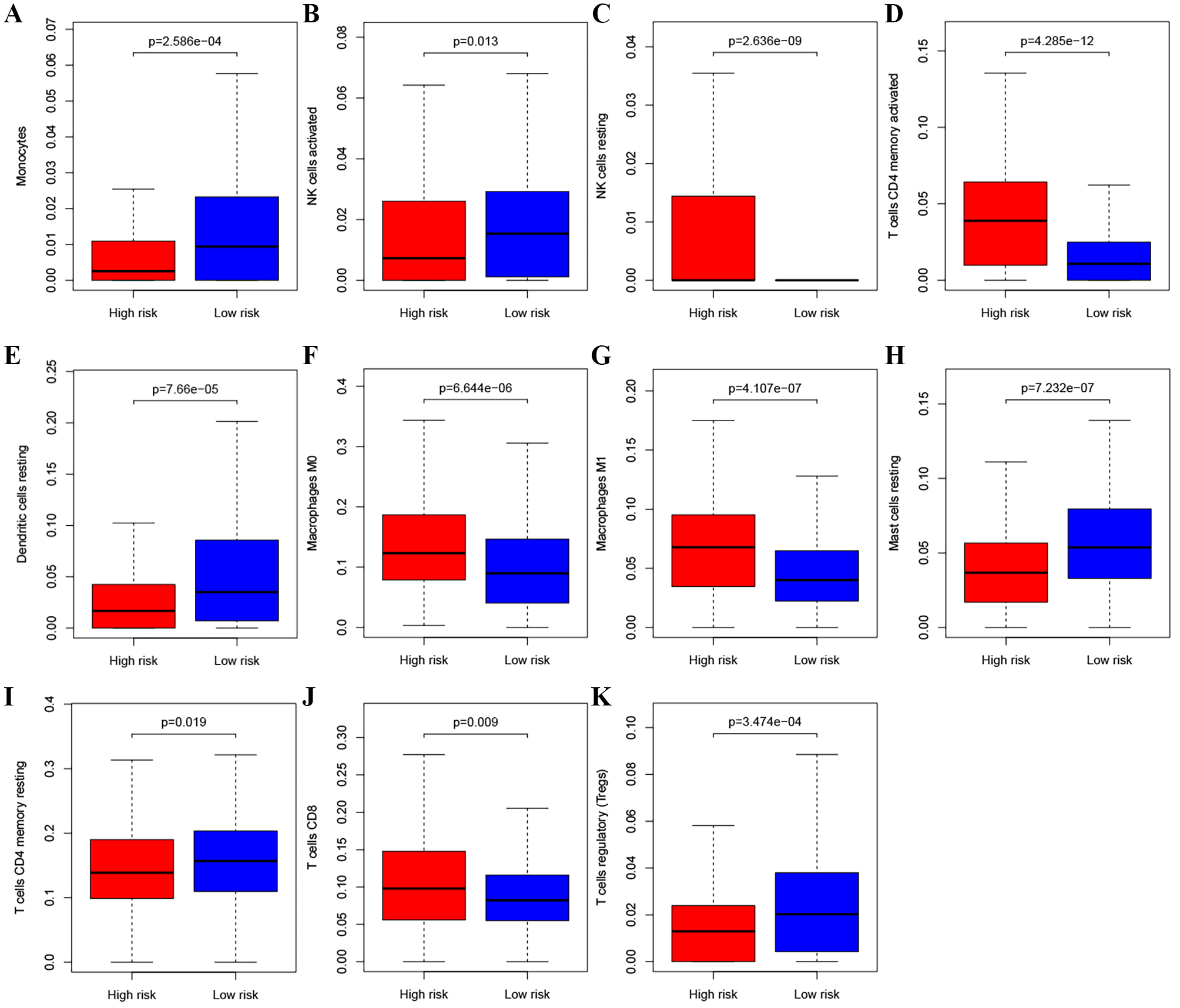

Supplement: Supplementary Figure 1 — Differences in immune-infiltrating cells between high- and low-risk patients. [file Image_1.TIF]
