# Supplementary material for: A Combined Two-mRNA Signature Associated With PD-L1 and Tumor Mutational Burden for Prognosis of Lung Adenocarcinoma
Source: Front Cell Dev Biol. 2021 Jan 26;9:634697. doi: 10.3389/fcell.2021.634697 (PMC7875126; doi:10.3389/fcell.2021.634697)
Supplement: Supplementary Table 1 — Relationship between signature genes and transcription factors. [file Table_1.DOCX]

**Table S1:** Relationship between signature genes and transcription factors.

| **Genes** | **TFs** | **cor** | **pvalue** | **Regulation** |
| --- | --- | --- | --- | --- |
| **ANLN** | BACH1 | 0.352327611 | 9.1E-16 | postive |
|  | BRCA1 | 0.678047571 | 3.01E-67 | postive |
|  | CBX3 | 0.491415846 | 3.75E-31 | postive |
|  | CBX7 | -0.513097173 | 2.94E-34 | negative |
|  | CDK2 | 0.551459283 | 2.48E-40 | postive |
|  | CDK8 | 0.354083507 | 6.41E-16 | postive |
|  | CENPA | 0.625480717 | 1.47E-54 | postive |
|  | DNMT1 | 0.453119943 | 3.51E-26 | postive |
|  | E2F1 | 0.441420412 | 8.76E-25 | postive |
|  | E2F3 | 0.429252982 | 2.19E-23 | postive |
|  | E2F6 | 0.336514157 | 1.94E-14 | postive |
|  | E2F7 | 0.541687295 | 1.04E-38 | postive |
|  | EED | 0.422730117 | 1.16E-22 | postive |
|  | ELK1 | 0.300411574 | 1.12E-11 | postive |
|  | EP400 | 0.31247057 | 1.47E-12 | postive |
|  | EZH2 | 0.536045265 | 8.49E-38 | postive |
|  | FOS | -0.307503076 | 3.43E-12 | negative |
|  | FOSL1 | 0.449167239 | 1.06E-25 | postive |
|  | FOXA2 | -0.430005537 | 1.8E-23 | negative |
|  | FOXK1 | 0.390405926 | 2.74E-19 | postive |
|  | FOXM1 | 0.661652291 | 5.15E-63 | postive |
|  | H2AFX | 0.482848656 | 5.51E-30 | postive |
|  | HCFC1 | 0.317763231 | 5.87E-13 | postive |
|  | HDAC2 | 0.429429308 | 2.09E-23 | postive |
|  | HIF1A | 0.400546296 | 2.63E-20 | postive |
|  | HNF1B | -0.345247249 | 3.66E-15 | negative |
|  | JMJD6 | 0.345637262 | 3.39E-15 | postive |
|  | KDM1A | 0.338980237 | 1.22E-14 | postive |
|  | LIN9 | 0.471633384 | 1.66E-28 | postive |
|  | LMNB1 | 0.5799447 | 2.23E-45 | postive |
|  | MYBL2 | 0.552346691 | 1.76E-40 | postive |
|  | MYH11 | -0.303073002 | 7.21E-12 | negative |
|  | NCAPG | 0.692340018 | 3.6E-71 | postive |
|  | POLR2B | 0.311855123 | 1.64E-12 | postive |
|  | POLR3A | 0.348887389 | 1.8E-15 | postive |
|  | POLR3G | 0.425939113 | 5.14E-23 | postive |
|  | PRKDC | 0.4913144 | 3.87E-31 | postive |
|  | SAP30 | 0.326031621 | 1.34E-13 | postive |
|  | SMC1A | 0.41094309 | 2.18E-21 | postive |
|  | SMC3 | 0.455623996 | 1.73E-26 | postive |
|  | SSRP1 | 0.500559173 | 1.96E-32 | postive |
|  | STAT1 | 0.398665155 | 4.08E-20 | postive |
|  | STAT6 | -0.340050239 | 9.95E-15 | negative |
|  | SUMO2 | 0.341203866 | 7.98E-15 | postive |
|  | TCF21 | -0.324450645 | 1.79E-13 | negative |
|  | TEAD1 | 0.363234728 | 9.95E-17 | postive |
|  | TEAD4 | 0.481670759 | 7.93E-30 | postive |
|  | TFAP2A | 0.366166271 | 5.41E-17 | postive |
|  | TRIM28 | 0.332433748 | 4.16E-14 | postive |
|  | TTF2 | 0.535558741 | 1.02E-37 | postive |
|  | WDR5 | 0.370781714 | 2.05E-17 | postive |
|  | YY1 | 0.315608211 | 8.55E-13 | postive |
| **ARNTL2** | BACH1 | 0.360950948 | 1.59E-16 | postive |
|  | BRCA1 | 0.361527619 | 1.42E-16 | postive |
|  | CBX7 | -0.311468311 | 1.75E-12 | negative |
|  | CDK2 | 0.334863061 | 2.65E-14 | postive |
|  | CENPA | 0.315029163 | 9.46E-13 | postive |
|  | E2F7 | 0.441677847 | 8.18E-25 | postive |
|  | EED | 0.311680377 | 1.69E-12 | postive |
|  | ETS1 | 0.357156303 | 3.45E-16 | postive |
|  | FOSL1 | 0.526626613 | 2.6E-36 | postive |
|  | FOSL2 | 0.328292739 | 8.91E-14 | postive |
|  | FOXA2 | -0.351725629 | 1.03E-15 | negative |
|  | FOXM1 | 0.447778536 | 1.55E-25 | postive |
|  | HIF1A | 0.417322813 | 4.53E-22 | postive |
|  | HNF1B | -0.356421825 | 4.01E-16 | negative |
|  | IRF1 | 0.423908054 | 8.63E-23 | postive |
|  | KDM5A | 0.30884381 | 2.73E-12 | postive |
|  | NCAPG | 0.417004053 | 4.9E-22 | postive |
|  | PML | 0.374974269 | 8.34E-18 | postive |
|  | POLR3G | 0.375083254 | 8.15E-18 | postive |
|  | RARG | 0.374796697 | 8.67E-18 | postive |
|  | SMAD3 | 0.359215594 | 2.27E-16 | postive |
|  | SNAI2 | 0.391093319 | 2.34E-19 | postive |
|  | STAT1 | 0.491370511 | 3.8E-31 | postive |
|  | TEAD4 | 0.400002753 | 2.98E-20 | postive |
